# Lightweight Transformer-based knowledge distillation framework for high-dimensional spatiotemporal radiomics in breast cancer risk prediction

**DOI:** 10.1186/s42492-026-00231-3

**Published:** 2026-09-03

**Authors:** Haofan Huang, Han Zhou, Kaibin Huang, Jie Yang, Peixi Ying, Puxiang Lai, Yan Lin, Yi Gao

**Affiliations:** 1 School of Biomedical Engineering, Shenzhen University Medical School, Shenzhen University, Shenzhen, 518060 China; 2 Department of Biomedical Engineering, The Hong Kong Polytechnic University, Hong Kong, SAR China; 3 Radiology Department, Second Affiliated Hospital of Shantou University Medical College, Shantou, Guangdong Province 515041 China; 4 Department of Ophthalmology, Zhujiang Hospital of Southern Medical University, (The Second Clinical Medical College), Guangzhou, Guangdong Province 510282 China

**Keywords:** Breast cancer, Spatiotemporal radiomics, Tumor heterogeneity, Deep learning, Knowledge distillation, Precision medicine

## Abstract

Breast cancer exhibits significant spatiotemporal heterogeneity. Traditional radiomics approaches usually rely on low-temporal-resolution imaging and discrete image phases, failing to capture the rapid and continuous kinetic evolution within the tumor microenvironment. Although ultrafast dynamic contrast-enhanced magnetic resonance imaging captures precise contrast agent permeation, current methodologies lack systematic, dedicated computational frameworks to extract these high-dimensional dynamic features. To bridge this gap, this study developed and validated a robust two-stage spatiotemporal radiomics framework for high-dimensional feature selection and predictive modeling. First, a novel gradient-dynamics-based feature selection algorithm was designed to robustly identify highly discriminative kinetic trajectory patterns and overcome the dimensionality curse of time-series data. Second, a lightweight Transformer-based predictive network incorporating a self-attention knowledge distillation mechanism was deployed to capture deep representational knowledge and enhance the differentiation between benign and malignant lesions. Experimental results demonstrated that the proposed framework outperformed traditional baseline models using delta features or pharmacokinetic parameters, achieving an area under the curve of 0.959 ± 0.022 and an accuracy of 92%, an 8% improvement over conventional temporal radiomics models (*P* < 0.05). Furthermore, through the visualization of attention heat maps, the model provides highly interpretable evidence of the critical enhancement phases and spatiotemporal evolutionary patterns that drive model decisions. This framework introduces a viable, visually interpretable, and quantitative tool for characterizing tumor heterogeneity, demonstrating its potential to assist in personalized clinical decision-making for breast cancer.

**Supplementary Information:**

The online version contains supplementary material available at https://doi.org/10.1186/s42492-026-00231-3.

## Introduction

Breast cancer is the most prevalent malignancy among women worldwide and imposes a substantial public health burden owing to its high incidence and mortality rates [1]. In clinical practice, the heterogeneity of the condition makes accurate diagnosis and personalized treatment essential. The accurate assessment of tumor progression and prediction of biological behavior are fundamental for the development of an optimal treatment plan. Although conventional breast imaging modalities such as ultrasound and mammography play a vital role in initial screening, they still face challenges in differentiating complex lesions and evaluating the intricate microvascular characteristics within the tumor [2].

In recent years, dynamic contrast-enhanced magnetic resonance imaging (DCE-MRI) has emerged as a pivotal modality for precise diagnosis of breast cancer. Continuous scans can noninvasively capture the entire kinetic process of contrast agent perfusion and diffusion within a tumor, thus acquiring rich pathophysiological information. However, conventional low-temporal-resolution (> 60 s) sequences used in clinical practice primarily focus on static morphological features and late-stage enhancement curves. Owing to the restricted sampling frequency, these sequences cannot adequately capture the transient exchange process of contrast agents within the tumor microenvironment, leading to an inherent loss of information regarding early perfusion characteristics and microvascular permeability [2, 3]. In contrast, the ultrafast DCE-MRI sequence (approximately 5 s) used in this study offers irreplaceable incremental value. It precisely delineates the ultra-early kinetic patterns of contrast agents as they transition from the vasculature to interstitial space [4–6]. This enables the quantitative characterization of blood perfusion and osmotic pressure within the tumor microenvironment. By integrating advanced radiomic feature extraction techniques, we can leverage these high-temporal-resolution data to extract dynamic time-series features, which can more effectively characterize such complex biological behaviors. In comparison with static morphological indicators, these features can reveal spatiotemporal heterogeneity within tumors in a more sensitive and comprehensive manner, thereby improving diagnostic accuracy in identifying highly invasive lesions.

Despite the rich information inherent in high temporal resolution radiomic features, current research still faces several limitations: (1) Most studies typically extract features from only two or three predefined “representative phases” (e.g., precontrast, peak, and delayed phase), failing to exploit the dimensional advantages of high-frequency time series [7–11]. Such discrete time points struggle to accurately capture continuous evolutionary patterns, which limits both the diagnostic performance and depth of biological interpretation of existing models. (2) There is a lack of specialized screening and modeling frameworks for these high-dimensional dynamic features, leaving a vast number of potentially significant temporal biomarkers that are systematically unexplored and unvalidated.

To bridge these research gaps, this study aimed to develop and validate a framework for the screening and predictive analysis of radiomic features in high-temporal-resolution sequences. The core objectives of this framework are: (1) to construct a feature selection algorithm suitable for such high-dimensional time-series data, aiming to robustly identify the most discriminative enhanced trajectory patterns from massive dynamic information, and (2) to build predictive models based on these high-dimensional time-series radiomic features to more accurately distinguish between benign and malignant breast tumors and to conduct a more detailed assessment of their invasiveness. Through this research, we hope to not only more comprehensively characterize the degree of tumor progression but also provide strong quantitative evidence for discovering novel radiomics biomarkers related to tumor evolution, thereby providing a deeper radiological perspective for understanding the heterogeneity of breast cancer.

## Methods

### Study dataset

This retrospective study was approved by the Institutional Review Board, and all participants provided written informed consent. This study included a previously described subset of patients (*n* = 86) [12], derived from a dataset of 96 patients who underwent breast MRI at a medical center in China between August 2018 and November 2021. All examinations were performed on a 3.0T MRI scanner (GE Medical System, Milwaukee, WI, USA) using the standard MRI acquisition protocol, including T1-weighted imaging (T1WI), T2-weighted imaging (T2WI), DCE-MRI, conventional diffusion-weighted imaging, and diffusion kurtosis imaging, for multiparametric MRI radiomics analysis. In this study, only the original DCE-MRI sequences were used for analysis; the detailed imaging parameters are summarized in Table 1. The gadolinium contrast agent (Jiangsu Hengrui Pharmaceuticals Co., Ltd., Jiangsu, China) was intravenously administered at a dose of 0.2 mmol/kg body weight and at a flow rate of 2 mL/s. Of the initial 96 patients, 10 were excluded due to the absence of original DCE-MRI sequences. Consequently, a final cohort of 86 patients with 114 breast lesions were included.

**Table 1:** Imaging parameters for dynamic contrast-enhanced magnetic resonance imaging

Parameter	Dynamic contrast-enhanced magnetic resonance imaging
Sequence	VIBRANT
Repetition time (ms)	3.9
Echo time (ms)	2.1
Field of view (cm)	35
Fat suppression	SPECIAL
Matrix	256$\:\times\:$256
Bandwidth (Hz/pixel)	83.3
Total scan time (s)	326
Acquired images	K^trans^ map, K_ep_ map, V_e_ map

### Image annotation and pre-processing

Images were evaluated by three radiologists, each with more than 5 years of experience in breast MRI. Histopathological results for all cases were independently assessed by three histopathologists with 6 years of experience. Lesion segmentation was performed using 3D Slicer (version 4.11.0), and radiomic features were extracted using the PyRadiomics platform. For each of the 114 samples, 1130 distinct radiomics features were independently extracted from 64 temporal phases of the DCE-MRI sequences. Consequently, the final dataset encompassed 1130 features per phase across 64 time points for 114 breast lesions.

### Model development

**Fig. 1:**
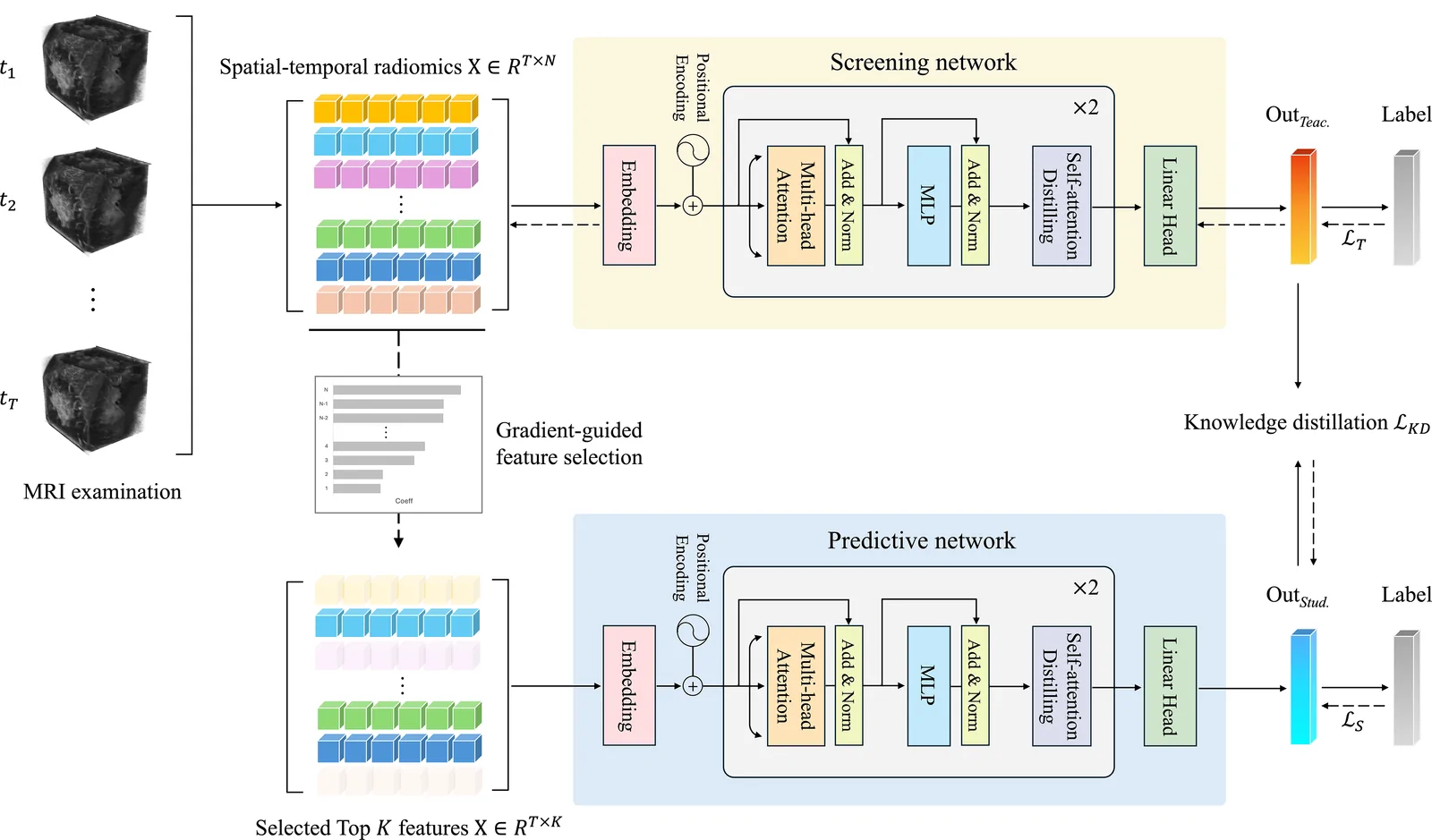
Network framework diagram. The screening network identifies Top-K robust features by tracking temporal gradient sequences. The predictive network then leverages knowledge distillation to transfer representational knowledge from the feature selection stage, collaboratively optimizing diagnostic performance

The proposed framework is shown in Fig. 1 and was designed as a two-stage algorithmic pipeline.

#### Robust feature selection based on gradient dynamics

The screening network received high-temporal-resolution radiomic features as input. During the training process, the gradient for each feature was defined as the partial derivative of the loss function of the feature-screening network with respect to its corresponding feature weight. The gradient values generated across all epochs were recorded to construct the feature gradient sequence, denoted as $\:{G}_{i}=\left\{{g}_{1},{g}_{2},\dots\:,{g}_{E}\right\}$, where * E* represents the total number of training iterations. Subsequently, customized dynamic stability and trend consistency weighting algorithms were applied to this sequence to quantify the quality of each feature.

1. Volatility assessment: The intensity of gradient fluctuations was measured by calculating the average rolling variance $\:{\sigma\:}^{2}$ within a sliding window, with the window size set at 5 in this study. The stability score was defined as ${{\cal S}_{tab}} = 1/\left( {{\sigma ^2} \cdot \lambda } \right)$, where $\:\lambda\:$ is a scaling factor set to 10^3^. Lower volatility indicates that the model’s learning process for the corresponding feature is more convergent, reflecting higher stability.
2. Trend consistency assessment: The gradient sequence was divided into overlapping windows, and a univariate linear regression $\:{g}_{i}\approx\:{\alpha\:}_{j}\cdot\:{x}_{i}+{\beta\:}_{j}$ was performed on each window to calculate the gradient trend term $\:{k}_{j}=\mathrm{s}\mathrm{g}\mathrm{n}\left({\alpha\:}_{j}\right)$. Based on these trend terms, the frequency of the trend reversals between adjacent windows ($\:{N}_{chang}$) was calculated. The consistency score was then defined as $\>{{\cal C}_{ons}} = 1 - {N_{chang}}/\left( {M - 1} \right)$, where * M* denotes the total number of windows (* M=6* in this study). The consistency quantifies the deflection frequency of the feature gradient in the macroscopic direction. A higher score indicates that the feature is more consistent in the macroscopic optimization direction.
3. Composite scoring and selection: The final importance score for each feature was defined as the product of its stability and consistency scores: $\:{S}_{final}={\mathcal{S}}_{tab}\times\:{\mathcal{C}}_{ons}$. All features were then ranked in descending order based on their $\:{S}_{final}$ values. The top-K robust features with the highest scores were selected as the optimized input for the subsequent predictive modeling stage.

Unlike screening methods such as L1 regularization or pointwise statistical tests, which decompose time-series features into independent time nodes for analysis, our method directly analyzes the dynamic change patterns of features across the entire time series. This allows us to perceive the overall dynamic behavior of spatiotemporal features, thereby preserving their complete information structure in the time dimension and enabling the global mining of complex spatiotemporal radiomic features.

#### Knowledge distillation-based predictive network

The selected robust temporal features were used to train the predictive network to distinguish between benign and malignant breast tumors. Given the small sample size of the cohort dataset, a knowledge distillation architecture [13] was introduced at this stage to optimize the learning process. Specifically, the screening network from the first stage serves as the Teacher Model, whereas the predictive network serves as the Student Model. Both screening and predictive networks use a lightweight Transformer framework comprising only two layers of multihead attention modules [14, 15]. By transferring the deep representational knowledge captured by the Teacher Model within the feature space, the Student Model was guided through collaborative training, thereby strengthening its ability to discriminate malignant lesions. Furthermore, a self-attention distillation mechanism was designed to capture and enhance key temporal features, facilitating efficient processing of the input time series. The distillation process is formulated as: $\:{\mathrm{X}}_{j+1}^{t}=\mathrm{M}\mathrm{a}\mathrm{x}\mathrm{P}\mathrm{o}\mathrm{o}\mathrm{l}\left(\mathrm{G}\mathrm{E}\mathrm{L}\mathrm{U}\left(\mathrm{N}\mathrm{o}\mathrm{r}\mathrm{m}\left(\mathrm{C}\mathrm{o}\mathrm{n}\mathrm{v}1\mathrm{d}\left({\mathrm{X}}_{j}^{t}\right)\right)\right)\right)$, where $\:\mathrm{C}\mathrm{o}\mathrm{n}\mathrm{v}1\mathrm{d}\left(\cdot\:\right)$ performs one-dimensional convolutional filtering across the temporal dimension, followed by Batch Normalization (Norm) and the GELU activation function [16, 17]. The training loss function is the weighted loss of the distillation and cross-entropy losses.

### Statistical analysis

The comparative models followed a standardized experimental workflow using LassoCV for automated feature selection, combined with GridSearchCV for hyperparameter optimization to ensure optimal model performance. Multiple metrics were used to evaluate the predictive performance, including the area under the receiver operating characteristic curve (AUC), accuracy, sensitivity, specificity, precision, and F1 score. Calibration curves were used to assess the agreement between predicted and observed frequencies. Furthermore, a visual interpretation of the model’s focus and discrimination process regarding the spatiotemporal evolution patterns of key features was provided through the distribution of attention weights. To ensure a fair and accurate comparison between the different algorithms, five runs of fivefold cross-validation were performed, with all cross-validation folds generated using the same random seed. The model training and evaluation were conducted in Python using the PyTorch (v2.4.0), scikit-learn (v1.6.1), and scipy (v1.13.1) packages. Statistical differences in the predictive performances of the models were evaluated using a paired t-test. The Benjamini-Hochberg procedure was applied to correct for multiple comparisons. A two-sided *P* < 0.05 was considered statistically significant.

## Results

### Patient characteristics

A total of 86 patients (median age, 41 years [IQR: 33–50 years]) who underwent DCE-MRI were included in this study. Of the 114 lesions, 44 (39%) were pathologically confirmed as malignant and 70 (61%) as benign. In the malignant group, triple-negative breast cancer (TNBC) and histological Grade III tumors accounted for 7 (16%) and 10 (23%) cases, respectively. Within the framework of fivefold cross-validation, the dataset in each fold was partitioned into a training set (80%) and a validation set (20%) using a stratified random sampling approach to ensure equal representation of malignant and benign lesions across both groups. An oversampling strategy was incorporated into the training pipeline to mitigate the potential classification bias induced by class imbalance. Crucially, to prevent data leakage and ensure unbiased evaluation, the oversampling process was strictly applied to the training split within each cross-validation fold, whereas the validation split retained its original distribution for final performance testing.

### Selected features

**Fig. 2:**
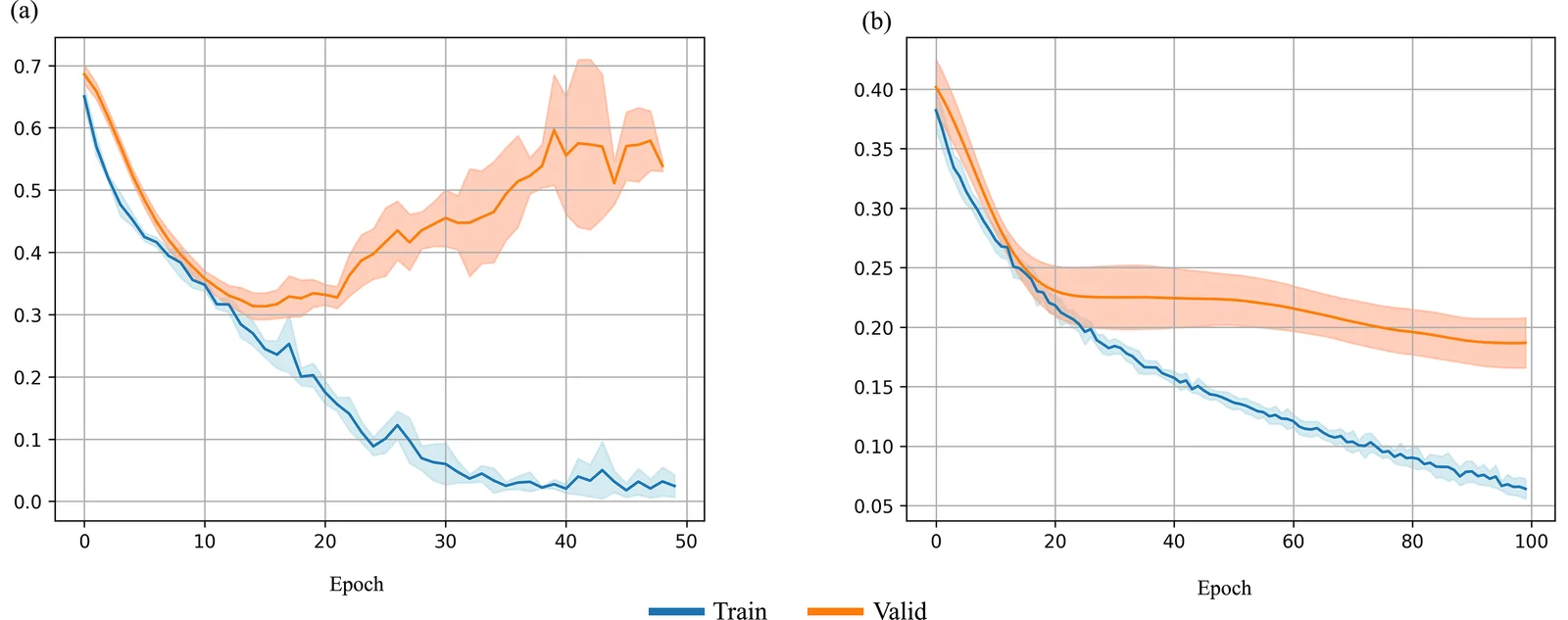
Cross-entropy loss curves (*y*-axis) for the feature screening network (**a**) and the predictive network (**b**). The feature screening network triggers early stopping at its optimal convergence point (Epoch 20) for subsequent gradient-based feature scoring. Driven by the optimized feature subset, the predictive network exhibits smoothly decreasing loss trajectories, demonstrating stable regularized convergence that effectively mitigates overfitting and oscillations on the small-scale dataset

In the feature selection stage, we recorded the dynamic gradient information of all features during the iteration process. The validation loss curve was employed as a monitoring metric to mitigate noise interference and overfitting associated with high-dimensional inputs. As illustrated in Fig. 2a, the network reached its optimal fitting state at the 20th epoch, after which signs of overfitting emerged. Consequently, we extracted the gradient sequences before this overfitting point to calculate the final importance score for each feature. To strike a balance between computational efficiency and prediction performance, we screened the 21 highest-ranking core features (approximately 2%). This feature selection pipeline was executed independently across all 25 cross-validation runs. Within each run, the gradient computations and feature rankings were derived exclusively from the training split, whereas the validation split served solely as a regularization monitor for the early stopping mechanism and did not participate in any parameter updates. Figure 2b shows that the training and validation loss curves of the predictive network exhibited a smooth decline. This trend suggests that incorporating knowledge distillation and robust feature selection as regularization methods allows the model to function efficiently on a small-scale dataset, preventing training oscillations and overfitting. Table 2 lists the selected spatiotemporal features from the best-performing run (peak AUC), including five original features, two features based on the Laplacian of Gaussian (LoG) transform, and 14 features based on the wavelet transform. Importantly, to ensure the generalizability of the feature profile and eliminate potential bias from a single split, we included the mean importance scores and standard deviations (SD) of these features across all 25 cross-validation cycles. The results demonstrated that the core features preferred by the optimal model maintained high and stable weights across all data partitions, further confirming their key clinical diagnostic value for breast cancer risk prediction.

**Table 2:** Selected feature subset with importance scores and stability statistics

Selected feature	$\:{S}_{final}$
ST1_original_shape_Maximum3DDiameter	0.675$\:\pm\:$0.100
ST2_original_shape_MinorAxisLength	0.659$\:\pm\:$0.102
ST3_original_shape_SurfaceArea	0.837$\:\pm\:$0.107
ST4_original_shape_SurfaceVolumeRatio	0.840$\:\pm\:$0.086
ST5_original_shape_VoxelVolume	0.862$\:\pm\:$0.074
ST6_log-sigma-3-0-mm-3D_glszm_SmallAreaLowGrayLevelEmphasis	0.773$\:\pm\:$0.109
ST7_log-sigma-5-0-mm-3D_glszm_SmallAreaLowGrayLevelEmphasis	0.609$\:\pm\:$0.093
ST8_wavelet-LLH_firstorder_Kurtosis	0.643$\:\pm\:$0.113
ST9_wavelet-LLH_gldm_DependenceNonUniformityNormalized	0.629$\:\pm\:$0.106
ST10_wavelet-LLH_ngtdm_Busyness	0.698$\:\pm\:$0.126
ST11_wavelet-LHL_glszm_LargeAreaHighGrayLevelEmphasis	0.651$\:\pm\:$0.046
ST12_wavelet-LHL_glszm_SmallAreaLowGrayLevelEmphasis	0.615$\:\pm\:$0.050
ST13_wavelet-LHH_firstorder_Kurtosis	0.699$\:\pm\:$0.076
ST14_wavelet-HLL_glszm_SmallAreaLowGrayLevelEmphasis	0.650$\:\pm\:$0.062
ST15_wavelet-HLL_gldm_SmallDependenceLowGrayLevelEmphasis	0.621$\:\pm\:$0.064
ST16_wavelet-HLH_firstorder_Kurtosis	0.653$\:\pm\:$0.095
ST17_wavelet-HLH_glszm_LargeAreaLowGrayLevelEmphasis	0.685$\:\pm\:$0.066
ST18_wavelet-HHL_glszm_LargeAreaEmphasis	0.590$\:\pm\:$0.053
ST19_wavelet-HHL_glszm_LargeAreaHighGrayLevelEmphasis	0.694$\:\pm\:$0.078
ST20_wavelet-HHL_glszm_LargeAreaLowGrayLevelEmphasis	0.611$\:\pm\:$0.092
ST21_wavelet-HHH_glszm_GrayLevelNonUniformityNormalized	0.573$\:\pm\:$0.037

### Evaluation of diagnostic performance

To validate the superiority of the proposed framework, a comprehensive comparison was performed with the modeling approaches commonly used in current DCE-MRI analyses. These models were constructed using XGBoost and support vector machine (SVM) algorithms, with their primary distinction residing in the representation and processing of temporal radiomic features. Specifically, the XGBoost-1 and SVM-1 models were built based on radiomic features extracted from pharmacokinetic parameter maps (K^trans^, K_ep_, and V_e_ maps), whereas the XGBoost-2 and SVM-2 models were developed using delta radiomics features. Furthermore, we introduced baseline models (XGBoost-B and SVM-B) that feed full temporal raw radiomic features directly into a conventional machine learning pipeline. This aims to eliminate the experimental bias induced by input information asymmetry.

**Fig. 3:**
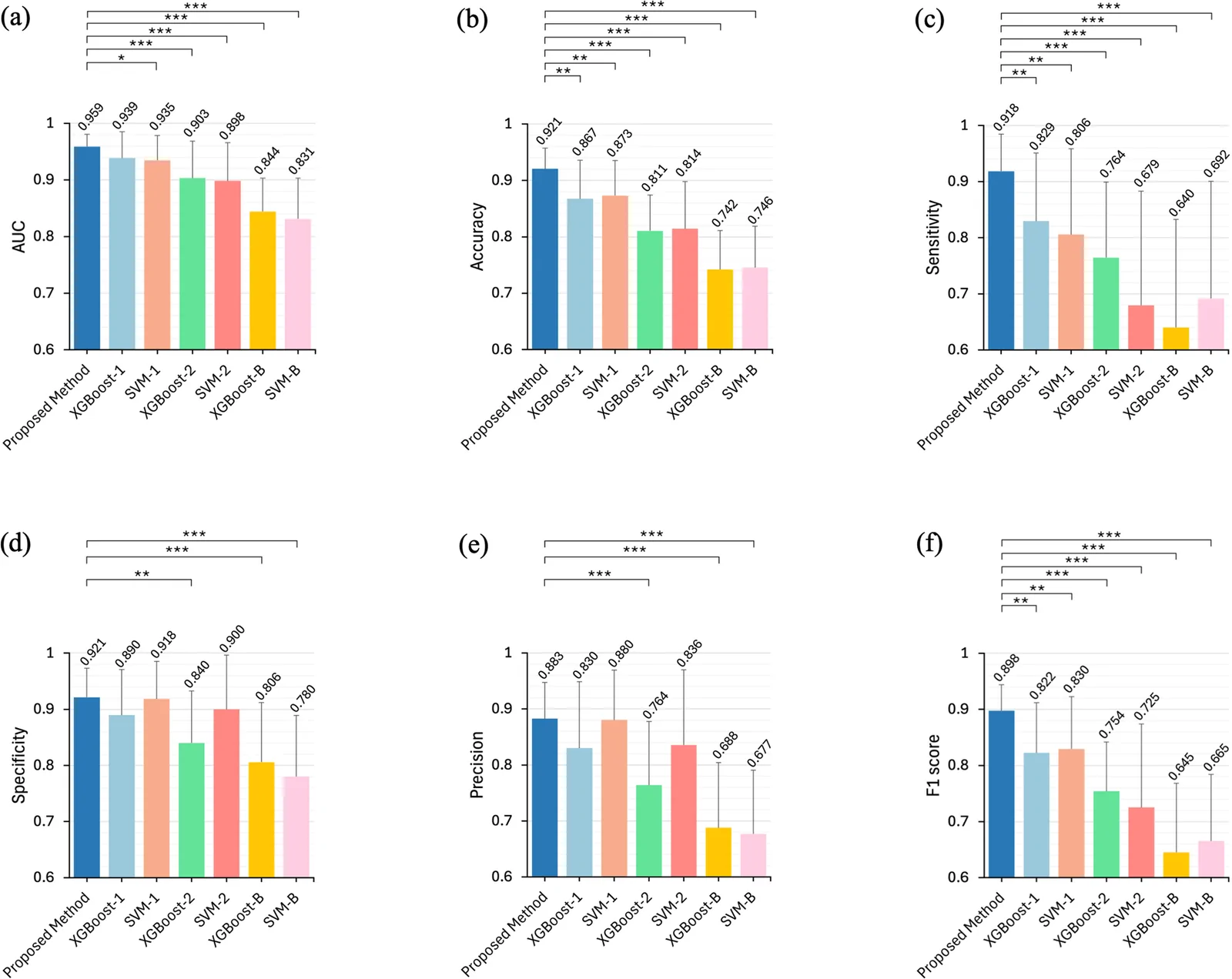
Performance evaluation results of each model. Bar graphs illustrate the (**a**) AUC, (**b**) accuracy, (**c**) sensitivity, (**d**) specificity, (**e**) precision, and (**f**) F1 score of each model evaluated on the independent validation set. ^*^P < 0.05, ^**^P < 0.01, ^***^P < 0.001. AUC: Area under the curve

Figure 3 illustrates the performance distribution of all models in the validation set. The experimental results show that the proposed method significantly outperforms the comparative models across most evaluation metrics, with lower standard deviations for each metric. Specifically, the proposed method achieved an AUC of 0.959$\:\pm\:$0.022 (95%CI, 0.951–0.967), with accuracy, sensitivity, specificity, precision, and F1 score reaching 0.921$\:\pm\:$0.037 (95%CI, 0.906–0.935), 0.918$\:\pm\:$0.066 (95%CI, 0.896–0.944), 0.921$\:\pm\:$0.052 (95%CI, 0.902–0.941), 0.883$\:\pm\:$0.064 (95%CI, 0.857–0.909), and 0.898$\:\pm\:$0.046 (95%CI, 0.879–0.917], respectively. In comparison, the XGBoost-1 approach yielded an AUC of 0.939$\:\pm\:$0.047 (95%CI, 0.920–0.955), accuracy of 0.867$\:\pm\:$0.068 (95%CI, 0.841–0.894) (*P* < 0.01), sensitivity of 0.829$\:\pm\:$0.122 (95%CI, 0.787–0.879) (*P* < 0.01), specificity of 0.890$\:\pm\:$0.081 (95%CI, 0.860–0.919), precision of 0.830$\:\pm\:$0.119 (95%CI, 0.785–0.879), and F1 score of 0.822$\:\pm\:$0.089 (95%CI, 0.791–0.857) (*P* < 0.01). The SVM-1 recorded an AUC of 0.935$\:\pm\:$0.044 (95%CI, 0.917–0.949) (*P* < 0.05), accuracy of 0.873$\:\pm\:$0.062 (95%CI, 0.849–0.897) (*P* < 0.01), sensitivity of 0.806$\:\pm\:$0.153 (95%CI, 0.748–0.861) (*P* < 0.01), specificity of 0.918$\:\pm\:$0.067 (95%CI, 0.891–0.944), precision of 0.880$\:\pm\:$0.089 (95%CI, 0.847–0.912), and F1 score of 0.830$\:\pm\:$0.093 (95%CI, 0.794–0.866) (*P* < 0.01). The XGBoost-2 achieved an AUC of 0.903$\:\pm\:$0.065 (95%CI, 0.875–0.927) (*P* < 0.001), with accuracy, sensitivity, specificity, precision, and F1 score of 0.811$\:\pm\:$0.064 (95%CI, 0.788–0.834) (*P* < 0.001), 0.764$\:\pm\:$0.135 (95%CI, 0.709–0.812) (*P* < 0.001), 0.840$\:\pm\:$0.093 (95%CI, 0.803–0.874) (*P* < 0.01), 0.764$\:\pm\:$0.114 (95%CI, 0.727–0.813) (*P* < 0.001), and 0.754$\:\pm\:$0.088 (95%CI, 0.718–0.788) (*P* < 0.001), respectively. The SVM-2 recorded an AUC of 0.898$\:\pm\:$0.067 (95%CI, 0.873–0.923) (*P* < 0.001), accuracy of 0.814$\:\pm\:$0.083 (95%CI, 0.784–0.846) (*P* < 0.001), sensitivity of 0.679$\:\pm\:$0.204 (95%CI, 0.603–0.752) (*P* < 0.001), specificity of 0.900$\:\pm\:$0.097 (95%CI, 0.857–0.934), precision of 0.836$\:\pm\:$0.134 (95%CI, 0.787–0.889) and F1 score of 0.725$\:\pm\:$0.149 (95%CI, 0.673–0.784) (*P* < 0.001). The XGBoost-B achieved an AUC of 0.844$\:\pm\:$0.059 (95%CI, 0.824–0.864) (*P* < 0.001), with accuracy, sensitivity, specificity, precision, and F1 score of 0.742$\:\pm\:$0.069 (95%CI, 0.718–0.769) (*P* < 0.001), 0.640$\:\pm\:$0.192 (95%CI, 0.569–0.708) (*P* < 0.001), 0.806$\:\pm\:$0.106 (95%CI, 0.766–0.846) (*P* < 0.001), 0.688$\:\pm\:$0.117 (95%CI, 0.647–0.734) (*P* < 0.001), and 0.645$\:\pm\:$0.123 (95%CI, 0.597–0.694) (*P* < 0.001), respectively. The SVM-B recorded an AUC of 0.831$\:\pm\:$0.072 (95%CI, 0.803–0.860) (*P* < 0.001), accuracy of 0.746$\:\pm\:$0.073 (95%CI, 0.717–0.775) (*P* < 0.001), sensitivity of 0.692$\:\pm\:$0.209 (95%CI, 0.611–0.767) (*P* < 0.001), specificity of 0.780$\:\pm\:$0.109 (95%CI, 0.734–0.820) (*P* < 0.001), precision of 0.677$\:\pm\:$0.114 (95%CI, 0.632–0.718) (*P* < 0.001), and F1 score of 0.665$\:\pm\:$0.119 (95%CI, 0.621–0.707) (*P* < 0.001). These significant differences suggest that the proposed method is more precise for decoding complex dynamic evolutionary patterns within DCE-MRI sequences than the conventional delta features or pharmacokinetic parameters. This leads to superior predictive performance in differentiating benign and malignant breast lesions while demonstrating more robust and reliable generalization capabilities. The baseline models performed the poorest across all metrics because they treated time-series data as disconnected nodes or flattened arrays while lacking the handcrafted temporal information used in delta-feature or pharmacokinetic frameworks. Consequently, they failed to capture the evolutionary trajectory of the radiomics metrics. Owing to this poor performance, these models were excluded from subsequent profiling to maintain the focus of this study.

We also provided a visual presentation of the pooled out-of-fold performance in Fig. S1 to facilitate a comprehensive evaluation of the model’s overall discriminative capacity and clinical utility across the entire operational threshold and decision spectrum. Receiver operating characteristic (ROC) curve evaluation (Fig. S1a) shows that the proposed method has a consistently superior discriminative capability across the operational threshold range. The clinical decision curve analysis (DCA, Fig. S1b) shows that this framework provides a higher net benefit than all other benchmark pipelines across a broad range of decision thresholds. These results validate the effectiveness of our method in comprehensively mining and modeling high-dimensional spatiotemporal radiological features and demonstrate the clinical value of the model.

**Fig. 4:**
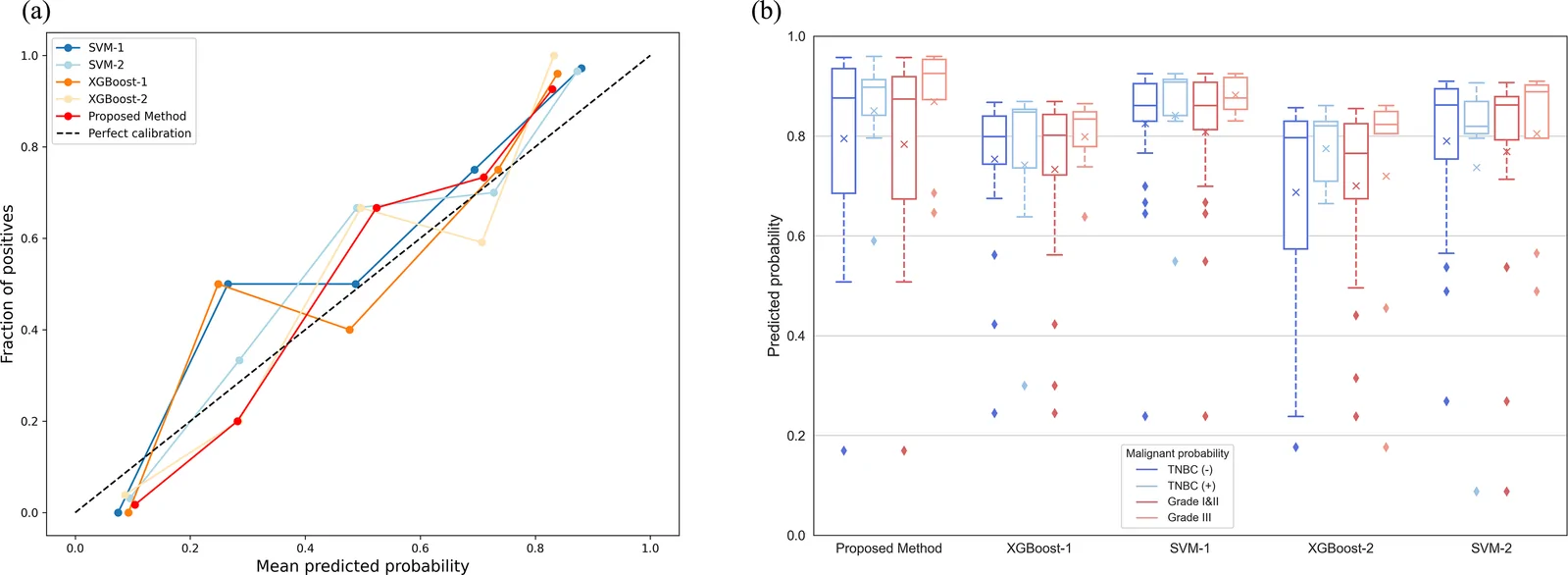
Calibration and subgroup analysis. (**a)** Calibration curves. The proposed model achieved better calibration, correcting biases across risk intervals. Brier score: proposed method = 0.081, XGBoost-1 = 0.147, SVM-1 = 0.125, XGBoost-2 = 0.170, SVM-2 = 0.154. (**b)** Boxplots of predicted probabilities by histological grade and molecular subtype. The proposed model yields higher scores for aggressive subtypes, whereas baseline models lack differentiation. TNBC: Triple-negative breast cancer; SVM: Support vector machine

Calibration curves (Fig. 4a) revealed that the XGBoost-1 and SVM-1 models exhibited overconfidence in the low-risk interval. The predicted malignancy probabilities were lower than the actual observed frequencies, indicating a notable underestimation of risk. In contrast, the proposed model aligned more closely with the ideal diagonal line, showing high consistency between the predicted malignancy probabilities and actual observations, suggesting the absence of over- or underfitting. This observation is strongly supported by the quantitative analysis of the Brier scores. Specifically, the proposed framework achieved the lowest Brier score of 0.081, outperforming the comparative models, including XGBoost-1 (0.147), SVM-1 (0.125), XGBoost-2 (0.170), and SVM-2 (0.154). This quantitative evaluation demonstrated that this framework has excellent calibration reliability and effectively alleviates the overconfidence bias in risk prediction.

### Subtype stratification and model interpretability

To further evaluate the sensitivity of the model to biological heterogeneity, stratification analyses were performed focusing on histological grades and TNBC subtypes. The box plots in Fig. 4b show that the predicted probabilities for the XGBoost-1, SVM-1, and SVM-2 models largely overlapped across different tumor grades. This demonstrates a high degree of homogeneity and the inability to effectively differentiate between well-differentiated and poorly differentiated tumors. In contrast, our method yielded higher predictive scores for histological Grade III and TNBC tumors, showing a capability for differentiating highly aggressive lesions. To elucidate the logic behind the model’s decision-making process for identifying lesions with distinct biological behaviors, three representative cases were selected: a benign cyst, an intermediate-grade invasive ductal carcinoma (TNBC−, grade II), and a high-grade invasive lobular carcinoma (TNBC+, grade III). A visualization analysis was conducted on the evolutionary trajectories and network attention weights of the 16 selected time-varying dynamic radiomic features in these examples.

**Fig. 5:**
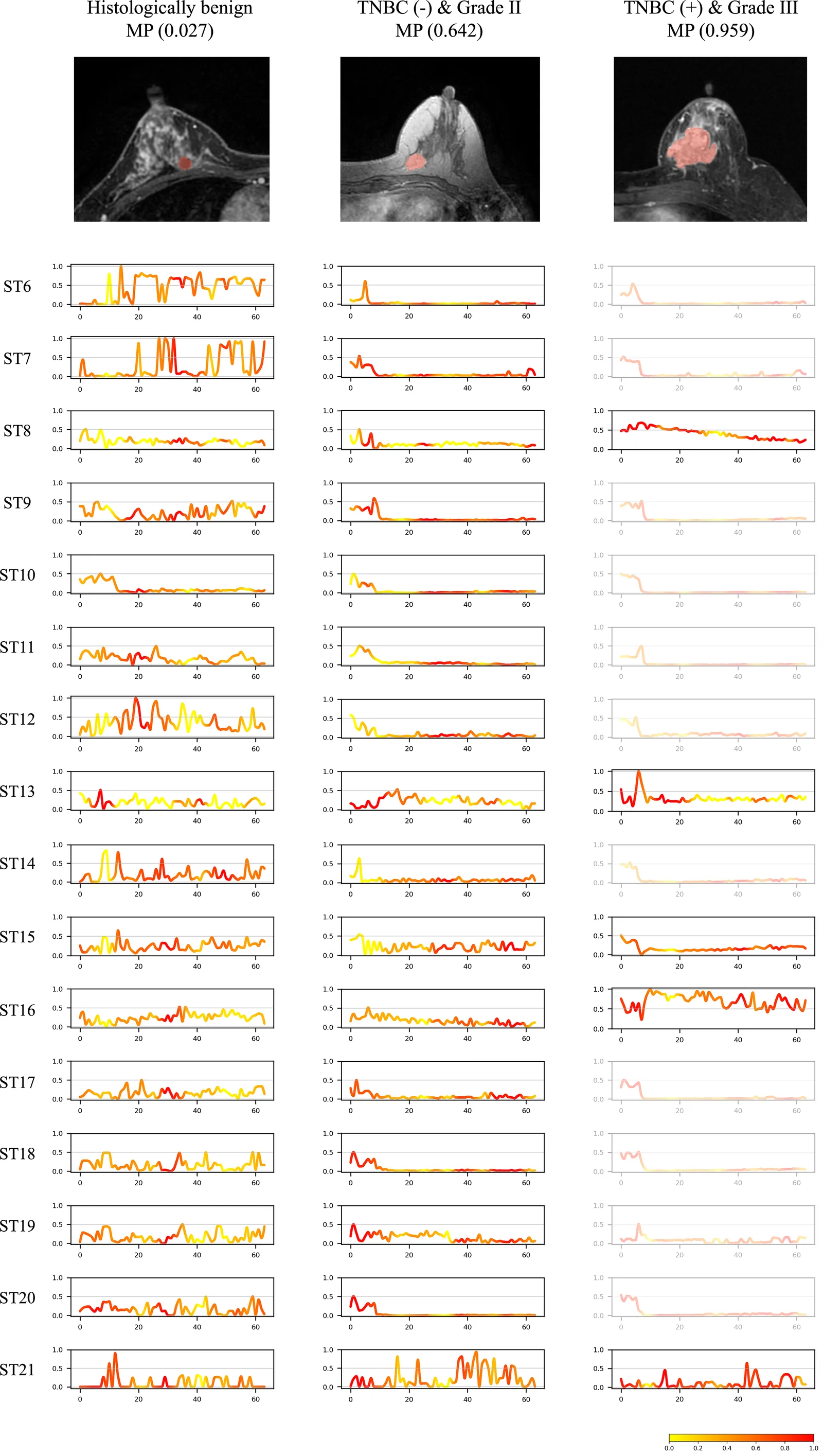
Temporal trajectories of dynamic radiomic features and corresponding network attention weights for representative cases. From left: benign cyst, moderately malignant invasive ductal carcinoma, and high-grade invasive lobular carcinoma. Curves depict feature value evolution over time, with colors indicating network attention weights. For the high-grade malignancy, features similar to those of the moderate malignancy are faded to highlight distinctive patterns. MP: Malignancy probability

As shown in Fig. 5, lesions with varying malignancy grades demonstrated distinct temporal feature patterns: (1) For benign cysts, most texture features extracted via wavelet and LoG filters exhibited persistent stochastic fluctuations throughout the dynamic enhancement process, without prominent unimodal peaks or abrupt shifts. Furthermore, kurtosis (representing the steepness of the gray-level distribution) within the lesions showed no directional changes over time. These patterns reflected the recurrent appearance and dissipation of enhancement signals, along with a lack of extensive contiguous high-intensity areas within the lesion. This is consistent with the pathophysiology of benign cysts [18–20], which typically feature mature vascular structures devoid of immature neovessels. This results in slow, homogeneous contrast agent inflow and clearance without rapid influx or washout. Therefore, the attention weights of the network were concentrated in the mid-term time window to capture periodic wall textures as primary discriminative evidence. (2) Intermediate-grade malignant tumors exhibit a biphasic evolutionary pattern. The feature signals peaked during the early arrival of the contrast agent, declined quickly, and remained stable at low levels during the mid-to-late phases. The enhancement characteristics in the early phase provide critical discriminative information, whereas late-phase washout kinetics also offer diagnostic value. Consequently, the attention weights of the network exhibited a bimodal distribution. Furthermore, similar to the kurtosis features of benign cysts, those of low-grade invasive ductal carcinomas exhibit relatively stable oscillations within a low range of values. Low-grade invasive ductal carcinomas usually retain glandular structures, and the tumor cells are arranged in a relatively orderly fashion [21]. This contrasts with high-grade malignancies, which have extremely heterogeneous areas. The network captures malignant characteristics while retaining benign-like features, thus providing an intermediate probability of malignancy. (3) In addition to demonstrating early enhancement characteristics, high-grade malignant tumors exhibited differentiated evolutionary patterns in their kurtosis features (ST8, ST13, and ST16) across various frequencies and orientations, providing rich information throughout the entire temporal sequence. During contrast perfusion, the spatial microstructures of invasive lobular carcinomas exhibit heterogeneous hemodynamic responses in different directions [22, 23]. These responses are captured by wavelet subbands that are sensitive to specific orientations and manifest as divergent temporal evolutions of the kurtosis features in different directions. The network captures this unique fingerprint using its attention mechanism, thereby yielding a higher probability of malignancy. Consequently, the network focuses primarily on the early phase while maintaining a secondary focus on the mid-to-late phases to track sustained or secondary variations in the kurtosis features.

## Discussion

This study proposes a screening and prediction framework for high-dimensional temporal features to address the current gap in DCE-MRI radiomic analysis. Previous studies either treated each time point in the DCE-MRI sequence as an independent node for separate evaluation or used only a few key time points. This greatly compromises the temporal resolution and fails to leverage the full potential of DCE-MRI sequences. This method distinguishes between benign and malignant lesions by comprehensively modeling the evolution of features at a high temporal resolution and the patterns of their spatiotemporal heterogeneity. The experimental results demonstrate that the proposed framework achieves an excellent diagnostic performance for breast lesions and successfully extracts biologically interpretable key imaging biomarkers from complex temporal data.

The proposed model achieved the best performance across all evaluation metrics, yielding an average AUC of 0.959$\:\pm\:$0.022, accuracy of 0.921$\:\pm\:$0.037, sensitivity of 0.918$\:\pm\:$0.066, specificity of 0.921$\:\pm\:$0.052, precision of 0.883$\:\pm\:$0.064, and F1 score of 0.898$\:\pm\:$0.046 on the validation set. Although previous studies have proposed the use of delta radiomics to characterize tumor evolution over time, such approaches only computed the numerical differences between specific discrete time points. This makes it difficult to characterize high-resolution temporal evolution patterns across an entire sequence, and this approach is sensitive to the choice of time points [23–25]. Quantitative pharmacokinetic parameters have shown potential for tracking tumor microenvironmental changes, but their accuracy is constrained by the number of scanning phases and temporal sampling frequency [26–28]. In contrast, the developed method effectively leverages the full-phase information of high temporal resolution DCE-MRI, enabling the precise capture of subtle hemodynamic evolution within tumors and their microenvironment. This in-depth mining of the spatiotemporal dimensions of the features endows the model with superior and more stable predictive performance, which significantly outperforms the comparison models in most evaluation metrics (*P* < 0.05). Importantly, the establishment of the baseline models eliminated the experimental bias stemming from input information asymmetry, thereby providing robust evidence that the proposed framework is the primary driver of performance enhancement. In addition, the ROC and DCA curves showed that the framework maintained superior differentiation across a wide range of thresholds and consistently delivered a higher net clinical benefit. Calibration curves and Brier scores demonstrated good agreement between the model-predicted probabilities and actual incidence, effectively correcting the overconfidence bias of the comparison models in low-risk intervals.

The present study further clarified the differences in the evolutionary patterns of temporal features among lesions of different pathological types. Since benign lesions typically lack the extensive neovascular network commonly found in malignant tumors, the inflow and washout of the contrast agent occur in a relatively balanced dynamic process [2, 18–20, 29]. Therefore, their temporal features exhibited persistent low-amplitude fluctuations, reflecting stable hemodynamics and lower vascular permeability. Malignant tumors typically exhibit rapid, early enhancement and washout patterns. This phenomenon is primarily attributed to abnormal hyperpermeability caused by dense neoangiogenesis, which leads to the rapid accumulation and subsequent swift clearance of contrast agents within a short period [2, 5, 20, 30–32]. Notably, owing to variations in the interstitial pressure and cellular heterogeneity across lesions of different malignant grades, kurtosis-based radiomic features demonstrate significant anisotropic dynamic differentiation. The highly disorganized microvascular architecture and dense cellular proliferation within high-grade malignancies drive more dramatic and irregular temporal variations in these radiomic features [20, 32], thereby providing the model with more discriminative spatiotemporal evolution information. Finally, through feature attention visualization, this study validated that the decision-making logic of the model was highly consistent with the hemodynamic patterns of tumors, significantly enhancing the model interpretability.

This study has several limitations. First, this was a single-center retrospective study with a relatively small sample size. In addition, there is a lack of suitable open-source datasets to serve as independent external validation sets, which, to some extent, constrains further evaluation of the model’s generalizability. All the performance metrics reported in this study were derived from validation splits. The validation split did not directly participate in gradient updates or parameter computations and was used to monitor the loss curve and trigger early stopping, providing indirect regularization information that influenced the training process. Thus, the primary numbers denote the localized cross-validation performance instead of an independent test benchmark. Future large-scale multicenter prospective studies are required to validate the robustness, generalizability, and clinical utility of this framework across different scanning devices and imaging protocols [33]. Second, the extraction and selection of full-temporal resolution features in this framework require more computational resources than conventional static radiomics approaches. To better adapt to the requirements of real-time clinical diagnosis, future efforts should focus on optimizing the algorithmic architecture to enhance inference efficiency while maintaining high temporal-resolution representation capabilities. Finally, this study relied solely on imaging data for modeling purposes. Future studies should integrate clinical indicators, pathological features, and genomic data to develop multiomics fusion models. By analyzing the relationship between imaging phenotypes and the underlying biological mechanisms, the predictive performance and clinical interpretability of the model in personalized precision medicine can be effectively improved.

## Conclusions

This study established a robust spatiotemporal radiomics framework that bridges the gap between high-frequency kinetic imaging and precise clinical decision-making in breast cancer. By leveraging the superior temporal resolution of ultrafast DCE-MRI, this study successfully moved beyond the traditional discrete-phase analysis to capture the continuous evolutionary trajectories of the tumor microenvironment. The two-stage methodology, integrating a novel gradient-dynamics-based feature selection algorithm with a Transformer-based knowledge distillation network, effectively overcomes the “curse of dimensionality” inherent in time-series data. The framework achieved a high diagnostic accuracy of 92.1% and an AUC of 0.959, significantly outperforming the conventional pharmacokinetic and delta-radiomics models. Beyond its predictive power, the integration of attention-based visual interpretability provides a window into the spatiotemporal heterogeneity of tumors, offering radiologists a quantitative tool for identifying high-risk pathologies. This research not only provides a sophisticated computational foundation for breast cancer risk assessment but also demonstrates the potential to support personalized clinical decision-making by transforming complex imaging sequences into actionable biological insights.

## Supplementary Information

Below is the link to the electronic supplementary material.

Supplementary Material 1

## Data Availability

Not applicable.

## Abbreviations

DCE-MRI: Dynamic contrast-enhanced magnetic resonance imaging
T1WI: T1-weighted imaging
T2WI: T2-weighted imaging
AUC: Area under the receiver operating characteristic curve
TNBC: Triple-negative breast cancer
LoG: Laplacian of Gaussian
SVM: Support vector machine
ROC: Receiver operating characteristic
DCA: Decision curve analysis
