# Supplementary material for: Lightweight Transformer-based knowledge distillation framework for high-dimensional spatiotemporal radiomics in breast cancer risk prediction
Source: Vis Comput Ind Biomed Art. 2026 Sep 3;9:20. doi: 10.1186/s42492-026-00231-3 (PMC13542040; doi:10.1186/s42492-026-00231-3)
Supplement: Supplementary file 1 [file 42492_2026_231_MOESM1_ESM.docx]

**Supplementary Information for “A lightweight Transformer-based knowledge distillation framework for high-dimensional spatiotemporal radiomics in breast cancer risk prediction”**

**Section S1. Supplemental Evaluation of Model Performance and Clinical Utility**

**Figure S1:** Pooled out-of-fold performance evaluation of different models. (A) Receiver operating characteristic (ROC) curves for comparing overall discriminative ability; (B) Clinical decision curve analysis (DCA) for quantifying net clinical benefits across running threshold probabilities. The solid colored lines represent the mean trajectories calculated across the cross-validation cycles, while the semi-transparent shaded bands denote the corresponding confidence intervals.
